# Functional role of aspartic proteinase cathepsin D in insect metamorphosis

**DOI:** 10.1186/1471-213X-6-49

**Published:** 2006-10-25

**Authors:** Zhong Zheng Gui, Kwang Sik Lee, Bo Yeon Kim, Yong Soo Choi, Ya Dong Wei, Young Moo Choo, Pil Don Kang, Hyung Joo Yoon, Iksoo Kim, Yeon Ho Je, Sook Jae Seo, Sang Mong Lee, Xijie Guo, Hung Dae Sohn, Byung Rae Jin

**Affiliations:** 1 College of Natural Resources and Life Science, Dong-A University, Busan 604-714, Korea; 2 Department of Agricultural Biology, National Institute of Agricultural Science and Technology, RDA, Suwon, Korea; 3 Department of Agricultural Biology, Chonnam National University, Gwangju, Korea; 4 School of Agricultural Biotechnology, Seoul National University, Seoul, Korea; 5 Division of Applied Life Science, Gyeongsang National University, Jinju, Korea; 6 Department of Life Science and Environmental Chemistry, Pusan National University, Miryang, Korea; 7 Sericultural Research Institute, Chinese Academy of Agricultural Sciences, Zhenjiang, China

## Abstract

**Background:**

Metamorphosis is a complex, highly conserved and strictly regulated development process that involves the programmed cell death of obsolete larval organs. Here we show a novel functional role for the aspartic proteinase cathepsin D during insect metamorphosis.

**Results:**

Cathepsin D of the silkworm *Bombyx mori *(*BmCatD*) was ecdysone-induced, differentially and spatially expressed in the larval fat body of the final instar and in the larval gut of pupal stage, and its expression led to programmed cell death. Furthermore, *BmCatD *was highly induced in the fat body of baculovirus-infected *B. mori *larvae, suggesting that this gene is involved in the induction of metamorphosis of host insects infected with baculovirus. RNA interference (RNAi)-mediated *BmCatD *knock-down inhibited programmed cell death of the larval fat body, resulting in the arrest of larval-pupal transformation. *BmCatD *RNAi also inhibited the programmed cell death of larval gut during pupal stage.

**Conclusion:**

Based on these results, we concluded that BmCatD is critically involved in the programmed cell death of the larval fat body and larval gut in silkworm metamorphosis.

## Background

Insect metamorphosis is a complex, highly conserved, and strictly regulated process of developmental events. Metamorphosis is triggered by the steroid hormone ecdysone in the absence of the sesquiterpenoid juvenile hormone and is carried out by self-destructive mechanisms of programmed cell death [1]. The developmental process of different larval tissues during metamorphic transformation showed that tissues such as the silk gland and gut are completely histolyzed [2-4], while other tissues such as fat body undergo reorganization with histolysis [5,6], and predetermined imaginal tissues differentiate and grow into organs and external structures [4,7].

The ecdysone-induced transcription factor Broad-Complex (BR-C) plays an important regulatory role in metamorphosis [8-14]. It is required for differentiation of adult structures as well as for the programmed death of obsolete larval organs during metamorphosis. The *Bombyx *BR-C RNAi disrupted the differentiation of adult compound eyes, legs and wings, and also perturbed the programmed cell death of larval silk glands [4].

Additionally, the *Bombyx *BR-C function uncovers the programmed cell death of larval fat body and larval gut during silkworm metamorphosis. It is still unclear what gene products function in the programmed cell death of larval fat body and/or larval gut. Therefore, we asked whether cathepsins are involved in the metamorphic events of silkworm because, to date, studies in insects reveal that cathepsins also participate in developmental processes [2,15-23]. Recently, a study has shown that the temporal activity profile of an aspartic proteinase is associated with fat body histolysis during *Ceratitis capitata *early metamorphosis [6]. Studies of insect cathepsins strongly implicate the involvement of activated proteinases in metamorphic events. Thus, it is of interest to know whether cathepsin has any functional roles in insect metamorphosis through a loss-of-function test.

Here, we have focused on cathepsin D, a lysosomal aspartic proteinase, as a metamorphosis-specific proteinase involved in metamorphic events. To help elucidate the molecular mechanisms of metamorphosis in the silkworm, we first cloned the *Bombyx mori *cathepsin D (*BmCatD*) gene from the silkworm. We examined the expression profile of *BmCatD *during development; *BmCatD *is induced by the steroid hormone ecdysone and baculovirus infection, and is expressed in a tissue- and developmental stage-specific pattern in the larval fat body of the final instar and in the larval gut of pupal stage. Finally, we demonstrate that loss of *BmCatD *function disrupts two classes of metamorphic events in *Bombyx*, larval-pupal transformation and programmed cell death of larval gut.

## Results and discussion

### A novel aspartic proteinase (BmCatD) gene cloned from the silkworm B. mori

**Figure 1:**
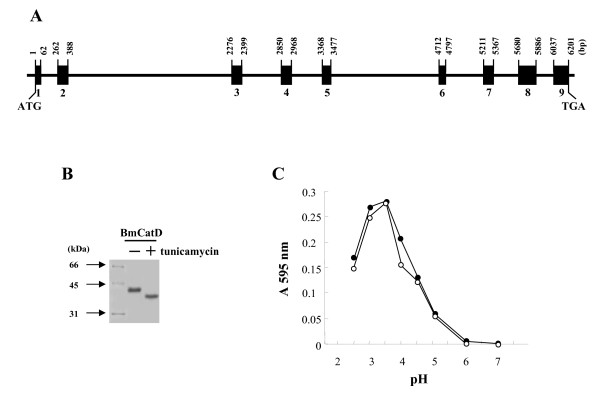
Characterization of *BmCatD *gene and protein product. (A) Genomic structure of *BmCatD *gene revealed by PCR amplification from *BmCatD *cDNA. Numbers indicate the position in the genomic sequences. GenBank accession numbers are AY297160 (*BmCatD *cDNA) and DQ417605 (*BmCatD *genomic DNA). (B) N-linked glycosylation of recombinant BmCatD expressed in baculovirus-infected insect Sf9 cells. The recombinant AcNPV-infected Sf9 cells were treated with (+) or without (-) tunicamycin and the cell lysates were analyzed by Western blot analysis. (C) Optimum pH of recombinant BmCatD. The N-linked glycosylated (solid circle) and nonglycosylated (open circle) BmCatD polypeptides were purified from culture supernatants. The pH dependency of recombinant BmCatD activity on 2% hemoglobin was assayed directly at different pHs.

The *BmCatD *cDNA was isolated by searching *B. mori *ESTs that encode a protein of 385 amino acids (GenBank accession number AY297160). Comparison of amplicon size between the genomic and cDNA sequences revealed the presence of nine exons and eight introns in *BmCatD *(Fig. 1A). The two catalytic centers and aspartic acid residues, as well as the six cysteine residues characteristic of aspartic proteinases [15,24,25], were conserved in BmCatD, indicating that BmCatD is a member of the same family as all other insect aspartic proteinases identified to date. BmCatD showed the closest amino acid identity with the aspartic proteinase of the mosquitoes *Anopheles gambiae *(64% identity) and *Aedes aegypti *(63% identity). However, this *BmCatD *gene did not align with any lepidopteran *CatD *gene identified to date.

Cathepsin D has been reported to be an N-glycosylated high mannose glycoprotein that functions as an acidic proteinase, with an optimal pH of 3.0 [6,15,26]. We found that recombinant BmCatD expressed in baculovirus-infected insect cells was N-linked glycosylated, but its N-linked glycosylation is not necessary for enzyme activity and that the purified recombinant BmCatD exhibited a high proteolytic activity at pH 3.0–3.5, establishing BmCatD as an aspartic proteinase (Fig. 1B,c).

### BmCatD is expressed in a developmental stage- and tissue-specific manner and its expression causes programmed cell death

**Figure 2:**
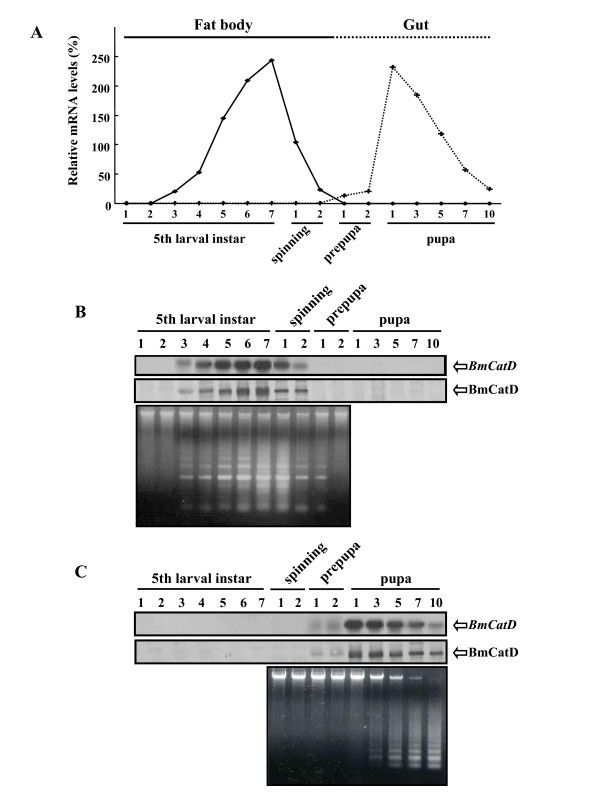
*BmCatD *expression profile and internucleosomal DNA fragmentation in the fat body and gut of *B. mori*. (A) Relative mRNA levels of *BmCatD *during the fifth larval instar to pupal stage. The level of *BmCatD *mRNA is calculated relative to that of the background (shown as 0%). The fat body and gut of *B. mori *were collected during the fifth larval instar to pupal stage (as indicated), respectively. (B, C) *BmCatD *expression and internucleosomal DNA fragmentation in the fat body (B) and gut (C) of *B. mori*. The expression level of *BmCatD *mRNA (upper) and its protein (middle) was analyzed by Northern blot and Western blot analyses, respectively. Internucleosomal DNA fragmentation in fat body and gut cells was assessed by 1.0% agarose gel electrophoresis (lower).

To examine the expression of *BmCatD *in various tissues during development, we first performed Northern blot analysis. We found that *BmCatD *was expressed in the fat body of the final (fifth) larval instar, but not in the pupal fat body, and was also seen in the gut during pupal development. Next, developmental stage- and tissue-specific profiles of *BmCatD *expression were performed using total RNA obtained from fat body and gut during the fifth larval instar to pupal stages (Fig. 2A). In the fat body, *BmCatD *mRNA was expressed from day 3 in the fifth larval instar to day 2 in the spinning stage (upper panel of Fig. 2B). On the other hand, *BmCatD *mRNA in larval gut was expressed during the entire pupal stage including prepupae (upper panel of Fig. 2C). The expression level of *BmCatD *was relatively low in the prepupal stage, increased dramatically on day 1 of the pupal stage, and thereafter gradually reduced but remained until adult eclosion. These results showed that the expression pattern of *BmCatD *was specifically started in the fat body on day 3 of the fifth larval instar and completely disappeared on day 1 of the prepupal stage. In larval gut, expression of *BmCatD *was precisely detected on day 1 of the prepupal stage, indicating that *BmCatD *is differentially and spatially expressed during development and its expression proceeds in a precise tissue- and developmental stage-dependent manner. Consistent with Northern blot data, the expression pattern of BmCatD protein, which was confirmed by Western blotting using the antibody of recombinant BmCatD that was expressed in baculovirus-infected insect cells, was observed in the fat body of the fifth larval instar (middle panel of Fig. 2B) and in the larval gut of pupal stage (middle panel of Fig. 2C).

The prospective fates of these tissues such as fat body and gut during metamorphic transformation are different. The larval gut is completely histolyzed during the pupal stage [2-4]; on the other hand, the fat body undergoes reorganization with histolysis during larval-pupal transformation [5,6]. It seems logical that the histolysis of larval fat body and larval gut represents a programmed cell death response. Therefore, we assayed whether the histolysis of larval fat body and larval gut is accompanied by internucleosomal DNA fragmentation that is a rapid and accurate indicator of the involvement of apoptosis in cell death [6,9]. To determine whether the *BmCatD *expression correlates with the histolysis of larval fat body and larval gut during metamorphosis, we analyzed the induction of programmed cell death in the fat body and gut tissues. Figure 2 also shows internucleosomal DNA fragments, seen as programmed cell death-specific laddering on agarose gel electrophoresis, for the fat body and gut during metamorphosis. The DNA fragmentation by histolysis of larval fat body was observed from day 3, peaked on day 7 in the fifth instar, and dramatically reduced on day 2 in the spinning stage (lower panel of Fig. 2B). In the larval gut, DNA fragmentation initiated on day 1 of the pupal stage and thereafter gradually increased until adult eclosion (lower panel of Fig. 2C). These results suggest that in fat body and gut, *BmCatD *expression was accompanied with DNA fragmentation. In addition, the DNA fragmentation rapidly and severely occurred in larval gut in the pupal stage. The result suggests that the developmental profiles of *BmCatD *expression, as judged by DNA fragmentation, differed between the larval gut, which undergoes programmed cell death during the pupal stage, and larval fat body, which survives into the adult phase but undergoes reorganization during larval-pupal transformation.

### BmCatD is induced by ecdysone and viral infection

**Figure 3:**
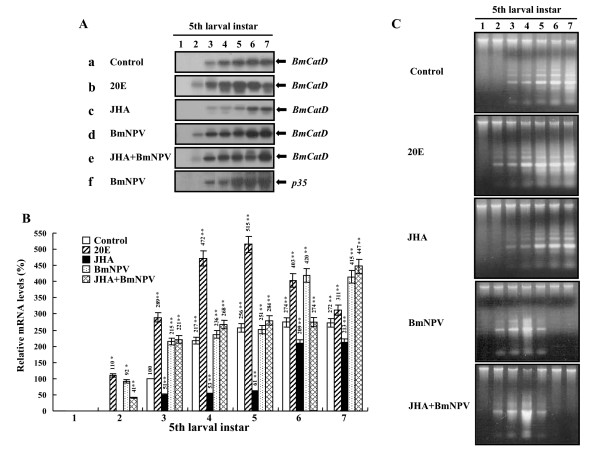
Hormonal and viral regulation of *BmCatD *mRNA expression. (A) Expression profiles of *BmCatD *mRNA in fat body of the fifth larval instar with hormonal and viral treatments. *B. mori *larvae on day 1 of the fifth instar were treated with 20E (b), JHA (c), BmNPV (d) or JHA+BmNPV (e), respectively. Total RNA from fat body was extracted at 1-day intervals post-treatment and analyzed by Northern blots. The control was the untreated larvae (a). BmNPV *p35 *(f) gene serves as a marker of viral infection. (B) Relative mRNA levels of *BmCatD *regulated by treatment. The levels of *BmCatD *mRNA are means of three assays, which are calculated relative to that of the expression recorded for the control (shown as 100%). Bars represent the means ± SE. Significance of the differences *versus *a control value is given by ** (*P *< 0.01) and * (*P *< 0.05). (C) Internucleosomal DNA fragmentation in the fat body of the fifth larval instar with hormonal and viral treatments. DNA from the fat body cells of all treated larvae was extracted at 1-day intervals post-treatment (as described in A), respectively. DNA fragmentation was assessed by 1.0% agarose gel electrophoresis.

Metamorphosis is regulated by changes in the titer of the steroid hormone ecdysone in the absence of the sesquiterpenoid juvenile hormone [1,27,28]. In order to determine if *BmCatD *expression is an ecdysone-triggered response, we examined effects of 20-hydroxyecdysone (20E) and juvenile hormone analogue (JHA) on *BmCatD *mRNA expression using the fat body of the fifth instar larvae. When 20E was injected into day 1 fifth instar larvae, *BmCatD *mRNA was clearly detected in the fat body on day 2 of the fifth larval instar (Fig. 3A-b), although no *BmCatD *expression was observed in the control during this period (Fig. 3A-a). On the other hand, topical application of JHA reduced *BmCatD *expression (Fig. 3A-c). These results show that *BmCatD *expression is up-regulated by 20E and down-regulated by JHA (Fig. 3B), implicating that these hormonal responses can explain ecdysone-induced expression of *BmCatD *in the fifth larval instar. Especially, 20E administration highly induced programmed cell death of larval fat body (Fig. 3C), indicating that the onset of larval fat body histolysis coincides with the expression of *BmCatD*. During insect metamorphosis, pulses of the steroid hormone ecdysone direct the destruction of obsolete larval tissues and the replacement of larval tissues and structures with adult forms [9]. Day 3 of the fifth larval instar in the silkworm is a critical period for larval growth, and during this period, prothoracic glands begin to secrete detectable ecdysteroid [27,28]. A pulse of ecdysone at the end of larval development triggers pupation, and the ecdysteroid activity induced by another ecdysone pulse again increases dramatically just before pupation [27,28]. The larval silk gland and larval gut begin programmed cell death in response to an increase in ecdysteroid concentration in the pupal stage [29-31]. Taken together, our results indicate that *BmCatD *is expressed exclusively in the larval fat body on day 3 of the fifth instar and in the larval gut on day 1 of the prepupal stage, and suggest that the expression of *BmCatD *is regulated in a tissue- and developmental stage-specific manner by ecdysone fluctuations.

Host insect infection with baculovirus induces metamorphosis [32,33]. This raises the possibility that BmCatD may be involved in the induction of metamorphosis of host insects infected with baculovirus. To examine this possibility, we carried out transcriptional induction analysis of *BmCatD *in baculovirus-infected silkworm larvae. When BmNPV was injected into larvae on day 1 of the fifth instar, *BmCatD *mRNA was detected in the fat body on day 2 of the fifth larval instar (Fig. 3A-d), although no *BmCatD *expression was observed in the controls (Fig. 3A-a). Consistent with this, the induced programmed cell death of larval fat body by viral infection was clearly observed on day 2 of the fifth larval instar (Fig. 3C). Furthermore, the level of *BmCatD *expression was found to be higher in virus-infected larvae than in uninfected controls (Fig. 3B). This result indicates that viral infection induced the *BmCatD *expression, which resulted in the induced programmed cell death in larval fat body, and suggests that *BmCatD *was involved in the induction of metamorphosis in the host insect infected with baculovirus. During virus replication, the prothoracic gland of host insects was observed to maintain characteristics indicative of ecdysone biosynthetic activities [33]. The haemolymph ecdysteroid level and prothoracic gland activity in baculovirus-infected larvae were higher than in controls, which continued until the late stages of viral infection, even during the time that controls had ceased to secrete ecdysone after molting [32,33]. Thus, these observations indicate that viral infection resulted in the up-regulation of *BmCatD*, as shown in 20E treatment, and suggest that *BmCatD *up-regulation is the result of alteration of host's hormonal system by viral infection.

To further understand the correlation between hormonal system and viral infection, we injected larvae on day 1 of the fifth instar with *Bombyx mori *nucleopolyhedrovirus (BmNPV) just after JHA treatment, which down-regulates *BmCatD *expression. Interestingly, in both JHA and BmNPV treatments, *BmCatD *expression was up-regulated in a viral infection-dependent manner (Fig. 3A-e), thus indicating that viral infection plays a role in the regulation of *BmCatD *expression, regardless of the presence or absence of JHA. The host's hormonal system, altered by viral infection, induces metamorphosis [32,33], which suggests that metamorphosis induction due to virus infection functions as a viral defense system of the host insect. In contrast, baculoviruses are known to contain the unique *p35 *gene, which blocks virus-induced apoptosis [34]. Therefore, the inhibition of DNA fragmentation at later stages of viral infection (Fig. 3C) was likely due to P35 of BmNPV. This result was consistent with previous observations indicating that baculovirus infection blocks the progression of fat body degradation [35].

### Loss of BmCatD function disrupts metamorphic events in the silkworm

**Figure 4:**
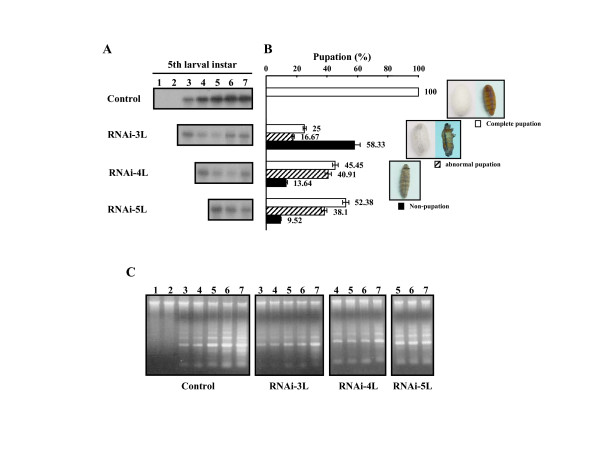
Effects of *BmCatD *RNAi on *B. mori *development. (A) *BmCatD *expression profile in RNAi-mediated *B. mori *larvae and controls. *BmCatD *dsRNA was injected into larvae on day 3 (RNAi-3L), 4 (RNAi-4L) and 5 (RNAi-3L) of the fifth instar, respectively. The control was the untreated larvae. Total RNA from the fat body was extracted at 1-day intervals post-treatment. The expression level of *BmCatD *mRNA was analyzed by Northern blot analysis. (B) Pupation rate in RNAi-mediated *B. mori *larvae and controls. The pupation rates are the means of three assays. Bars represent the means ± SE. (C) Internucleosomal DNA fragmentation of the fat body in RNAi-mediated *B. mori *larvae. DNA from the fat body cells of all treated larvae was extracted at 1-day intervals post-treatment (as described in A), respectively. DNA fragmentation was assessed by 1.0% agarose gel electrophoresis.

It is known that in the fat body of insects, the lysosomal system is involved in cellular remodeling, which is associated with metamorphosis and termination of egg maturation cycles [6,15,36,37]. However, there has been relatively little research into the substantial functional role of CatD in metamorphosis in insects, especially in *Lepidoptera*. Therefore, we next asked whether BmCatD is functionally involved in the metamorphosis of the silkworm, and whether silkworms have any defects in the *BmCatD *RNA interference (RNAi) process. In order to determine the functional role of BmCatD in silkworms during metamorphosis, we reduced the endogenous *BmCatD *mRNA levels in the fat body of silkworm larvae by using RNAi, and then confirmed the reduced *BmCatD *mRNA levels by using Northern blot hybridization. *BmCatD *mRNA levels in fat body of *BmCatD *RNAi-mediated silkworm larvae were significantly reduced compared to the control silkworm larvae (Fig. 4A). To characterize the functional role of BmCatD in silkworm larvae, it is important to obtain silkworm larvae containing little or no *BmCatD*. This approach relies on the degree of *BmCatD *reduction caused by RNAi, as a measure of RNAi function, in order to explore the proposed role of BmCatD in silkworm larvae.

In *BmCatD *RNAi-mediated silkworm larvae, larval-pupal metamorphosis was strongly affected by *BmCatD *RNAi. Compared to controls, all of which underwent regular larval-pupal transformation, three-fourths of *BmCatD *RNAi on day 3 fifth instar larvae arrested during larval-pupal metamorphosis (Fig. 4B). In this case, a large portion of *BmCatD *RNAi-mediated silkworm larvae was nonpupated (58.3%) and 16.7% of larvae were abnormally pupated. Similar effects were respectively seen in 55% and 48% of *BmCatD *RNAi on day 4 and 5 fifth instar larvae, and the abnormal pupation rate was relatively high in these cases (Fig. 4B). The arrest of larval-pupal transformation was observed in *BmCatD *RNAi-mediated silkworm larvae and indicated that BmCatD is necessary for the larval-pupal metamorphosis in the silkworm. It is formally conceivable that BmCatD might have additional functions in other developmental processes, and reduction in BmCatD via RNAi in these processes might directly or indirectly contribute to developmental arrest.

**Figure 5:**
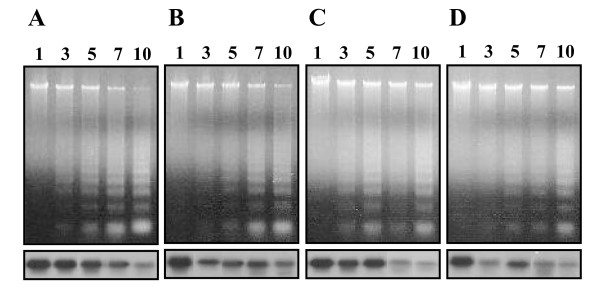
Internucleosomal DNA fragmentation of the larval gut in RNAi-mediated *B. mori *pupae. *BmCatD *dsRNA was injected into pupae on day 1 (B), 5 (C) or both 1 and 5 (D) of the pupal stage, respectively. The control was the untreated pupae (A). DNA was extracted from the gut on day 1, 3, 5, 7, and 10 of the pupal stage (as indicated), respectively. DNA fragmentation was assessed by 1.0% agarose gel electrophoresis (upper). Total RNA from larval gut was extracted as above. The expression level of *BmCatD *mRNA was analyzed by Northern blot analysis (lower).

We next tried to determine whether *BmCatD *could induce DNA laddering in larval gut during pupal development. To provide evidence that BmCatD is involved in programmed cell death of larval gut, we also reduced the endogenous *BmCatD *mRNA levels in the gut of silkworm pupal stage by using RNAi and then examined the pattern of DNA fragmentation in the larval gut. As observed from the Northern blots (lower panels of Fig. 5), *BmCatD *levels were reduced in the larval gut of *BmCatD *RNAi-mediated silkworm pupae. Compared to controls, which undergo rapid and severe DNA fragmentation in the larval gut from day 3 of the pupal stage, *BmCatD *RNAi-mediated silkworm pupae exhibited an inhibition of DNA fragmentation in larval gut (upper panels of Fig. 5). When *BmCatD *dsRNA was injected repeatedly into pupae on day 1 and 5 of the pupal stage, the inhibition of DNA fragmentation in larval gut was more clearly affected. These results indicate that loss of BmCatD causes a defect in internucleosomal DNA fragmentation of larval gut, pointing to an important role of BmCatD in programmed cell death of larval gut.

As judged from the observed effect of *BmCatD *RNAi during metamorphosis, all observations in this study provide strong evidence that BmCatD was involved in the histolysis of larval fat body and larval gut, demonstrating a functional involvement as a metamorphosis-specific lysosomal proteinase. Recently, most studies of molecular mechanisms of metamorphosis in silkworm have focused on the metamorphosis-specific transcriptional factor BR-C [4,38-40]. It has been shown that the *Bombyx *BR-C is expressed in an ecdysone-induced manner and is required for programmed cell death of larval silk glands, as well as for the differentiation of adult structures including compound eyes, legs, and wings [4]. By focusing our findings on the BmCatD, we have been able to explain metamorphosis-specific functions of BmCatD.

## Conclusion

The work provided here demonstrates the involvement of cathepsin D as a metamorphosis-specific proteinase in metamorphic events. This finding is important in that it sheds new light on the functional role of cathepsin D in silkworm metamorphosis. The metamorphic defects seen in the *BmCatD *RNAi-mediated silkworm, such as larval-pupal transformation arrest and programmed cell death inhibition, highlight an important functional role of BmCatD in metamorphic processes and provide a foundation for a better understanding of the molecular mechanisms of insect metamorphosis.

## Methods

### Experimental animals

Larvae of the silkworm, *Bombyx mori*, used in this study were F1 hybrid Baekok-Jam supplied by Department of Agricultural Biology, The National Institute of Agricultural Science and Technology, Korea. Silkworms were reared on fresh mulberry leaves at 25°C, 65 ± % relative humidity, and 12 h light: 12 h dark photoperiod. Spinning (wandering) occurred on day 7 of the fifth instar, and pre-pupation and pupation occurred 2 days and 3 days thereafter. The first days corresponding to the developmental stages of the fifth larval ecdysis, spinning, and pupation were designated as day 1 of the fifth larval instar, spinning, and pupal stage, respectively.

### Gene cloning

The *BmCatD *cDNA was cloned from a cDNA library using whole bodies of *B. mori *larvae [41]. The sequences were compared using the DNASIS and BLAST programs provided by the NCBI [42]. MacVector (ver. 6.5, Oxford Molecular Ltd) was used to align the amino acid sequences of CatD. Genomic DNA, extracted from the fat body of single *B. mori *larva using a Wizard Genomic DNA Purification Kit (Promega), was used for PCR amplification with oligonucleotide primers designed from the *BmCatD *cDNA sequences. The nucleotide sequence was determined as described previously [41].

### Protein analysis

A baculovirus expression vector system [43], using the *Autographa californica *nucleopolyhedrovirus (AcNPV) and an insect cell line Sf9, was employed for the production of recombinant BmCatD protein. Recombinant BmCatD purification, antibody preparation, and Western blot analysis were performed as described previously [44]. The loading volume of protein samples in all Western blot analyses was 5 μg/lane. Tunicamycin treatment was performed as described previously [45]. Aspartic proteinase activity of BmCatD was measured as described previously [6].

### RNA analysis

Total RNA was isolated as described [44]. Northern blot and its image analysis were performed as described previously [44]. The loading volume of total RNA in all Northern blot analyses was 5 μg/lane.

### DNA fragmentation assay

DNA fragmentation from larval fat body and larval gut was assayed using an Apoptotic DNA-Ladder Kit (Roche Applied Science, Germany) according to the manufacturer's protocols. DNA was analyzed on a 1.0% agarose gel and visualized by ethidium bromide staining.

### Hormonal treatment and viral injection

Twenty-hydroxyecdysone (20E, Sigma) was dissolved in distilled water and stored at -20°C until used. Twenty micrograms of 20E dissolved in 20 μl of distilled water was injected into *B. mori *larvae on day 1 of the fifth instar. Fifty nanograms of a juvenile hormone analogue, fenoxycarb (Sankyo, Japan), dissolved in 20 μl of acetone were applied topically to larvae with a micropipette along the dorsal midline. For viral infection, BmNPV [44,46] was suspended in TC100 medium. *B. mori *larvae on day 1 of the fifth instar were injected with 50 μl of a viral suspension (1.0 × 10^5 ^PFU/larva). The fat body from all treated larvae was collected at 1-day intervals post-treatment and washed twice with PBS. Total RNA and genomic DNA were isolated from the tissues as described above. For injection in experiments, larvae of *B. mori *were injected with a sample solution between the first and second abdominal segments with a microsyringe. Each assay was replicated three times based on three independent tissue preparations. For comparison of relative *BmCatD *mRNA levels, statistical analysis of images of Northern blots was performed with Tukey's pairwise comparison test. Results are shown as mean ± SE of three animals per group. Significant *P *values were obtained by Tukey's test.

### RNAi

The MEGAscript RNAi kit (Ambion) was used to generate double-stranded RNA (dsRNA) corresponding to nucleotides 226 to 741 of the *BmCatD *cDNA. T7 promoter sites were added to the PCR primers *BmCatD*-Fi (5'-GCGTAATACGACTCACTATAGGGAGACCGCAGTCGTTCAAGGTGGTA-3') and *BmCatD*-Ri (5'-GCGTAATACGACTCACTATAGGGAGAGAACTCCCAGTACGTGTCCCG-3'). PCR reactions were conducted to generate complementary templates with a single T7 promoter site. T7 RNA polymerase was used to transcribe single-stranded RNA (ssRNA) from each DNA template over 4 h at 37°C. *BmCatD *dsRNA was produced by mixing solutions containing equal amounts of complementary ssRNA, incubating at 75°C for 5 min, and allowing the solution to cool down to room temperature. DNA and ssRNA were removed from the solution by digestion with DNase I and RNase at 37°C for 1 h. The dsRNA was purified using purification cartridges provided in the kit and dsRNA was eluted with two successive 50 μl washings of pre-heated (95°C) 10 mM Tris-HCl (pH 7.0) containing 1 mM EDTA. Finally, total dsRNA was quantified from the A_260_. *BmCatD *dsRNA (≈1 mg ml^-1^) was injected into larvae or pupae of *B. mori *(injection volume ≈50 μl/individual) using a sterile needle. After injection, all individuals were covered with paraffin.

## Authors' contributions

ZZG and KSL carried out most of the experiments in this study. BYK and YDW participated in the RNAi and DNA fragmentation assays. YSC and YMC assisted with cell culture, protein purification and antibody production. PDK carried out silkworm rearing. HJY and HDS assisted with the physiological characterization of the silkworm. IK helped to draft the manuscript. YHJ helped with the baculovirus expression vector and protein expression. SJS, SML, and XG assisted with the analysis of results. BRJ was responsible for the experiment design, analysis and interpretation of data and writing of manuscript. All authors approved the final manuscript.
